# Physical inactivity 5–8 years after anterior cruciate ligament reconstruction is associated with knee-related self-efficacy and psychological readiness to return to sport

**DOI:** 10.1136/bmjsem-2023-001687

**Published:** 2023-11-14

**Authors:** Maja Stigert, Farshad Ashnai, Roland Thomeé, Eric Hamrin Senorski, Susanne Beischer

**Affiliations:** 1 Department of Health and Rehabilitation/Physiotherapy, Institute of Neuroscience and Physiology, Sahlgrenska Academy, University of Gothenburg, Gothenburg, Sweden; 2 Sportrehab Sports Medicine Clinic, Gothenburg, Sweden; 3 Department of Orthopaedics, Sahlgrenska University Hospital, Mölndal, Sweden

**Keywords:** knee, physical activity, rehabilitation

## Abstract

**Objectives:**

To investigate whether patient demographics and patient-reported outcomes (PROs), respectively, are associated with physical inactivity (PI) 5–8 years after primary anterior cruciate ligament reconstruction (ACLR).

**Methods:**

This case control observational study included individuals who had undergone primary ACLR between the ages of 15 and 65 years and had responded to PROs 18 months postoperatively. These individuals were asked to answer a questionnaire regarding their present level of physical activity (PA) at 5–8 years after ACLR. Patient-demographic data and results from the Knee injury and Osteoarthritis Outcome Score, the Knee Self-Efficacy Scale and the ACL Return to Sport (RTS) after Injury scale from 18 months after ACLR were extracted from a rehabilitation-specific register. Univariable logistic regression analyses were performed with PI (<150 min PA per week/≥150 min PA/week) as the dependent variable.

**Results:**

Of 292 eligible participants, 173 (47% women; mean±SD age = 31±11 years) responded to the PA questionnaire. In all, 14% (n=25; 28% women) were classified as physically inactive. Participants with lower levels of present and future self-efficacy, OR 1.35 (CI 1.05 to 1.72) and OR 1.20 (CI 1.12 to 1.45), and lower levels of psychological readiness to RTS, OR 1.19 (CI 1 to 1.43), at the 18-month follow-up, had higher odds of being physically inactive 5–8 years after ACLR. None of the patient demographic variables was able to predict PI.

**Conclusion:**

Lower levels of knee-related self-efficacy and psychological readiness to RTS, 18 months after ACLR, were associated with PI 5–8 years after surgery.

**WHAT IS ALREADY KNOWN ON THIS TOPIC**

- Patients who return to sport after anterior cruciate ligament (ACL) reconstruction have been reported to have a stronger psychological profile, including psychological readiness to return to sport, self-efficacy and motivation. However, after ACL reconstruction (ACLR), patients are less physically active in terms of daily step count and minutes of physical activity at 2 and up to 5 years post surgery, respectively, compared with non-injured matched controls. The knowledge of which individual, as well as the rehabilitation-specific outcomes that are associated with physical inactivity more than 5 years postoperatively, is limited.

**WHAT THIS STUDY ADDS**

- In individuals, older than 15 years of age, lower levels of knee-related self-efficacy and psychological readiness to return to sports, 18 months after ACLR, were associated with physical inactivity 5–8 years after surgery.

**HOW THIS STUDY MIGHT AFFECT RESEARCH, PRACTICE OR POLICY**

- The associations between physical inactivity and knee-related self-efficacy and psychological readiness to return to sport, respectively, suggest that psychological factors appear to be important in the long-term perspective. To facilitate the identification of individuals at risk of becoming physically inactive and hopefully prevent physical inactivity, healthcare professionals are recommended to evaluate and, if needed, enhance psychological factors throughout the rehabilitation.

## Introduction

Physical activity (PA) is a fundamental aspect of human life, defined as ‘any voluntary body movement that requires energy expenditure’. The human body is developed to engage in PA and movement and the benefits of PA, including reduced mortality and the incidence of chronic diseases such as type 2 diabetes, cardiovascular disease, falls, depression and various types of cancer,^1 2^ are well established.

A rupture of the anterior cruciate ligament (ACL) is one of the most common severe knee injuries in young athletes^3^ and often entails a period of reduced frequency and intensity of PA, as well as a change in the type of PA, as less knee-strenuous activities may be warranted in the rehabilitation following ACL reconstruction (ACLR).^4 5^ Furthermore, the prevalence of physical inactivity (PI) has been reported to increase from 18 months to 3–5 years following ACLR.^5^ PI is a worldwide health problem and the WHO ranks PI as one of the four leading risk factors for global mortality.^6^ In Sweden, the prevalence of PI among adults is 34%.^7^ As the negative effects of PI can be reversed if an individual starts being physically active (PyA),^6^ healthcare professionals need to be able to identify individuals that have or run an increased risk of becoming physically inactive (PyI).

Patients who return to sport (RTS) after an ACLR have repeatedly been reported to have a stronger psychological profile, including psychological readiness to RTS, knee-related self-efficacy and motivation.^8–10^ However, it is not known how these factors are associated with the level of participation in general PA several years after ACLR.

Patients with an ACL injury run a 4–10 times greater risk of developing symptomatic knee osteoarthritis (OA) compared with knee-healthy individuals.^11 12^ However, regular exercise and more symmetrical knee extension strength can prevent the development of symptoms of OA in patients after an ACLR.^13 14^ With the already high risk of developing symptomatic knee OA^11 12^ and a higher body mass index,^12 15^ it is arguably crucial to promote PA after ACLR in order to facilitate gaining or maintaining muscular strength. This is especially important, as patients have been reported to be less PyA in terms of daily step count^16^ and minutes of PA^17^ at 2–5 years post ACLR, respectively, compared with non-injured matched controls. In addition, almost 20% of patients do not participate in any sport 2–4 years following ACLR.^18^ The knowledge of which individuals that may risk, as well as which rehabilitation-specific outcomes are associated with, PI more than 5 years postoperatively is, however, limited.

### Aim

The aim of this study was to investigate whether patient demographics and patient-reported outcomes (PROs) are associated with not meeting the recommendations for PA 5–8 years after a primary ACLR.

## Method and materials

### Study design and setting

This register study was based on prospectively collected data from an ongoing ACL rehabilitation outcome register, Project ACL, and reported according to the recommendation of the Strengthening the Reporting of Observational Studies in Epidemiology statement.^19^ Participation in the Project ACL is voluntary; all patients are given written information and informed consent is obtained.

### Participants

Individuals registered in the Project ACL with an 18-month follow-up after ACLR were assessed for eligibility. For the present study, patients with a registered primary ACLR before 28 February 2016 and who were 15–65 years of age at the time of surgery were included. Individuals who reported a new ACL injury, or another injury in the lower extremity that was deemed to have affected their subjective knee function (as reported by the participant himself or the test administrator), or an injury or illness that was deemed to have affected their ability to be PyA at the 5-year to 8-year follow-up, were excluded.

### Procedure/data collection

Five to 8 years after their ACLR, participants who met the inclusion criteria were contacted through email and text messages. Contact was initiated with an email including a website address with the informed consent form and a questionnaire regarding the present level of PA during a normal week. The participants were also asked to report if they had sustained more than one ACL injury or if they suffered from another injury/condition that affected their present level of PA. In total, the eligible patients received six reminders, one by email, followed by three separate text message reminders and finally a phone call from one of the authors (MS). Patients lost to follow-up were compared, with respect to patient demographics, with the included participants.

### Outcomes

Dependent variable PI (yes/no) at the 5-year to 8-year follow-up after ACLR was used as the dependent variable. To quantify the frequency and duration of PA, a validated questionnaire created by the Swedish National Board of Health and Welfare was used^20^ (online supplemental appendix 1). In the questionnaire, participants were asked to report the total time, in minutes, of moderate and vigorous PA, respectively, during a normal week, on a categorical scale from (a) 0–30 min up to (g) > 300 min. The total time of moderate and/or vigorous PA that was used for further analysis was calculated by multiplying the total minutes of vigorous activity by two and adding the minutes of moderate activity.^20^ The result of this questionnaire was used to group the participants into PyI and PyA. Participants who reported a total time of less than 150 min/week according to the WHO^6^ were categorised as PyI.

10.1136/bmjsem-2023-001687.supp1 Supplementary data

### Independent variables

Demographic data, extracted from the Project ACL, with respect to participants’ sex, age at the time of ACL injury/reconstruction and anthropometrics, were used as independent variables to compare participants who met the recommendations for PA with those that did not. All demographic data were self-reported on registration in the Project ACL. In addition, participants reported their present body weight and height at every follow-up.

To describe the participants with respect to PROs, at a time when most participants were expected to have terminated their rehabilitation and returned to a lifestyle more similar to that before suffering an ACL injury,^21^ data from the 18-month follow-up after ACLR were used.

To assess self-reported knee function, symptoms and quality of life, four of five subscales on the Knee injury and Osteoarthritis Outcome Score (KOOS) were used.^22^ In the Project ACL, the subscale of *activities of daily living* is only answered preoperatively and at 1, 2 and 5 years following ACL injury/reconstruction and was therefore not included in this study. The four subscales used in the present study have been reported to have acceptable reliability (Intraclass Correlation Coefficient (ICC) 0.81–0.93),^22^ whereas conflicting results have been reported with regard to the content validity of the KOOS for use in patients with an ACL injury.^23^ The participants were instructed to answer the items on the KOOS with respect to their previous week, with standardised answers on a five-point Likert scale. Scores were calculated according to Roos *et al*,^22^ where 0=extreme symptoms and 100=no symptoms.

The Tegner Activity Scale (Tegner)^24^ was used to document the participants’ preinjury and present level of PA, respectively, by asking the participants to rate their knee-strenuous activities between levels 0 (=least knee-strenuous) and 10 (=most knee-strenuous). In this study, a modified version of the Tegner, which does not contain any ‘0’ value, was used.^25^ Furthermore, the modified version allows ‘recreational sports’ up to level 9 instead of level 7, as in the original Tegner. The original version of the Tegner has been reported to have acceptable test–retest reliability, with an ICC of 0.8 in patients with an ACL injury or ACLR.^26^

To assess knee-related self-efficacy, the 18-item version of the Knee Self-Efficacy Scale (K-SES_18_) was used.^25^ The K-SES_18_ is reported to have good validity and reliability for patients after ACL injury and ACLR (ICC 0.92),^25^ and consists of two subscales: *present* and *future knee self-efficacy,* consisting of 14 and 4 questions, respectively. Participants rate each item on an 11-point Likert scale, where 0 represents *not at all certain* and 10 *very certain*. The final score on each subscale was calculated as the sum of the item scores divided by the number of items and it was then used for further analysis.

To assess the participants’ psychological readiness to RTS, the ACL Return to Sport after Injury (ACL-RSI) scale was used.^27^ The questionnaire is a valid and reliable 12-item scale with items relating to three types of psychological response associated with the resumption of sports following athletic injury—emotions, confidence in performance and risk appraisal.^27^ The total score is the sum of all the items and ranges from 0 (=worst) to 120 (=best possible outcome).

### Statistics

Statistical analysis was performed using SAS/STAT software (SAS Institute). Descriptive statistics for participant demographics and outcomes were reported as the count and distribution (%) for categorical variables, as the median with minimum and maximum for ordinal variables and as the mean±SD for continuous variables. To compare demographic data between participants lost to follow-up and the included participants, a dropout analysis was performed. For between-group comparisons, the Mann-Whitney U test was used for non-parametric data, as well as parametric data, as it did not meet the assumptions of normal distribution. For dichotomous variables, Fisher’s exact test was used for between-group comparisons and the χ^2^ test was used for ordered categorical variables. To analyse the association between the independent variables and PI, univariable logistic regression analyses, adjusted for participants’ sex, were performed and presented as the OR with a 95% CI. The OR is the ratio of the odds of an increase in the predictor of one unit. For body weight, height, the KOOS and, for the ACL-RSI, the OR per ten units was used. The number of events, that is, the number of participants classified as being PyI, stratified by predefined groups, was presented for each of the independent variables. Cut-off scores for the KOOS, K-SES and ACL-RSI are based on reported values indicating specific patient outcomes. The cut-off scores for the KOOS, the K-SES and the ACL-RSI were determined based on reported values. These include the Patient Acceptable Symptom State of the KOOS,^28^ an indicator of an acceptable level of self-efficacy of 7 on the K-SES,^29^ and a suggested score of 56, below which individuals run a higher risk of not returning to their preinjury level of sport for the ACL-RSI.^30^

A multivariable regression was planned and a minimum of >30 PyI participants were estimated to be needed to build a multivariable model adjusted for participants’ sex, including two independent variables. All the tests were conducted at the 5% significance level. Accuracy was determined by the general rules of thumb of Hosmer *et al*.^31^

## Results

**Figure 1:**
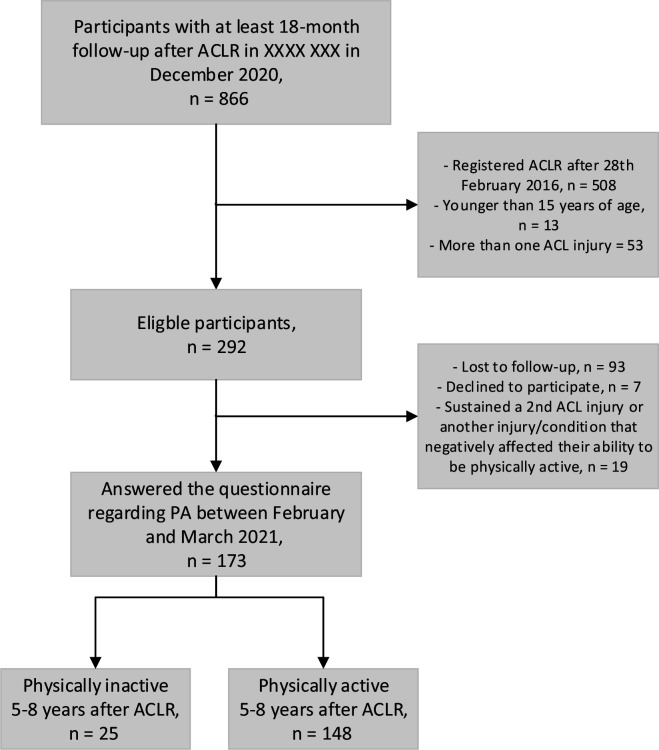
Flowchart of the inclusion process. ACLR, anterior cruciate ligament reconstruction; PA, physical activity.

A total of 292 participants were assessed to be eligible. Of them, 93 were lost to follow-up, 7 individuals declined to participate and 19 were excluded due to having sustained a second ACL injury or another injury or condition that negatively affected their ability to be PyA (figure 1). Finally, 173 participants (59%) of the 292 eligible participants answered the questionnaire regarding PA. Individuals lost to follow-up were younger at the time of their primary ACLR (difference in means: 5.7 years, p<0.001) and had a higher preinjury level of Tegner compared with the 173 participants in this study (table 1).

**Table 1:** Demographic data and drop-out analysis from the 18-month follow-up

	Lost to follow-up (n=93)	Total (n=173)	P value	Physically active* (n=148)	Physically inactive† (n=25)	P value
Female	40 (45%)	81 (47%)	0.606‡	74 (50%)	7 (28%)	0.066‡
BMI	23.8 (2.6)	24.2 (2.6)	0.049**§**	24.1 (2.4)	25.0 (3.2)	0.094§
Age at surgery	25.3 (8.3)	31.0 (11.3)	< 0.001**§**	30.8 (11.2)	32.2 (12.1)	0.56§
Tegner preinjury						
≤ 5	15 (19.2%)	44 (25.5%)		37 (25.0%)	7 (28%)	
6	8 (10.3%)	21 (12.1%)		18 (12.2%)	3 (12%)	
7	5 (6.4%)	32 (18.5%)		27 (18.2%)	5 (20.0%)	
8	18 (23.1%)	33 (19.1%)		29 (19.6%)	4 (16.0%)	
9	21 (26.9%)	34 (19.7%)		30 (20.3%)	4 (16.0%)	
10	11 (14.1%)	9 (5.2%)	0.009**¶**	7 (4.7%)	2 (8.0%)	0.58¶
Physical activity (minutes/week)	–	345 (0–540)		360 (150–540)	0 (0–135)	n.a.

For non-ordered categorical variables, n (%) is presented. For ordered categorical variables median (minimum-maximum) is presented. For continuous variables, the mean (SD) is presented.

P-value < 0.05 indicates a statistical significance.

*Physically active≥150 min/week.

†Physically inactive<150 min/week.

‡For comparisons between groups, Fisher’s exact test (lowest 1-sided p value multiplied by 2) was used for dichotomous variables.

§The Mantel-Haenszel χ^2^ test was used for ordered categorical variables.

¶Fisher’s non-parametric permutation test was used for continuous variables.

BMI, body mass index; n.a., not applicable; Tegner, Tegner Activity Scale.

The included participants answered the questionnaire regarding PA 5–8 years post ACLR (median: 6 years). The median minutes of PA in all the included participants (n=173) was 345 min/week (min 0, max 540), whereof 25 participants (14%) were classified as PyI (table 1). No differences in demographic characteristics were seen between PyA and PyI participants (table 1).

### Univariable logistic regression analyses

ORs and p values, adjusted for patients’ sex, for the independent variables are presented in table 2. Participants with lower levels of present and future knee-related self-efficacy (K-SES_18_ *present* OR (95% CI) 1.35 (1.05 to 1.72); p=0.017; K-SES_18_ *future* OR (95% CI) 1.20 (1.01 to 1.45); p=0.039) at the 18-month follow-up had higher odds of being PyI, 5–8 years after ACLR. Moreover, a lower psychological readiness to RTS at the 18-month follow-up was associated with PI (OR (95% CI): 1.19 (1.00 to 1.43); p=0.047) 5–8 years after ACLR.

**Table 2:** ORs associated with physical inactivity and univariable models adjusted for sex

	N	Value	N (%) of event of PI	OR (95% CI) PI	P value	Area under ROC curve (95% CI)
Body height (cm) (OR per 10 units)	173	154–<171	6 (12.8%)			
		171–<180	6 (8.7%)			
		180–198	13 (22.8%)	0.74 (0.37 to 1.45)	0.37	
Body weight (kg) (OR per 10 units)	172	50–<70	7 (11.1%)			
		70–<80	5 (9.3%)			
		80–117	13 (23.6%)	0.75 (0.50 to 1.12)	0.16	
BMI	172	<25.0	11 (9.6%)			
		25.0–30.0	12 (23.1%)			
		> 30.0	2 (40.0%)	0.92 (0.78 to 1.08)	0.29	
KOOS						
Symptoms (OR per 10 units)	173	32.0–<57.1	5 (21.7%)			
		> 57.1	20 (13.3%)	1.12 (0.86 to 1.47)	0.38	
Pain (OR per 10 units)	173	14.0–<88.9	10 (15.4%)			
		> 89.0	15 (13.9%)	1.28 (0.98 to 1.79)	0.13	
Sports (OR per 10 units)	173	0.0–<75.0	14 (17.9%)			
		> 75.0	11 (11.6%)	1.14 (0.95 to 1.37)	0.16	
QoL (OR per 10 units)	173	0.0–<62.5	12 (19.7%)			
		>62.5	13 (11.6%)	1.15 (0.93 to 1.41)	0.18	
K-SES						
Present	173	1–<7	7 (25.0%)			
		>7	18 (12.4%)	1.35 (1.05 to 1.72)	0.017	0.66 (0.54 to 0.78)
Future	173	1–<7	16 (23.2%)			
		>7	9 (8.7%)	1.20 (1.01 to 1.45)	0.039	0.67 (0.55 to 0.79)
ACL-RSI (OR per 10 units)	134	21–<56	12 (30.0%)			
		56–<77	3 (8.8%)			
		77–119	6 (10.0%)	1.19 (1.00 to 1.43)	0.047	0.66 (0.54 to 0.79)
Returned to previous level of Tegner (yes/no)	173	Yes	11 (13.8%)			
		No	14 (15.1%)	0.90 (0.38 to 2.17)	0.83	

All the tests were performed with univariable logistic regression. P values and OR and area under ROC curve (AUC) are based on original values and not on stratified groups. The OR is the ratio of the odds for an increase in the predictor of one unit. P value<0.05 indicates a statistical significance.

ACL-RSL, ACL-Return to Sport after Injury; BMI, body mass index; KOOS, Knee injury and Osteoarthritis Outcome Score; K-SES_18_, Knee Self-Efficacy Scale; PI, physical inactivity; ROC, receiver-operating characteristics; Tegner, Tegner Activity Scale.

The proportion of PyI participants was almost two times larger in the group of participants who scored below 57.1 on the subscale of *symptoms* on the KOOS and below seven on the *present* and *future* subscales on the K-SES_18_, compared with participants who scored above these cut-offs (table 2). Moreover, the proportion of PyI participants was 30%, that is, about three times larger in the group of participants who scored below the predefined cut-off score of 56 points on the ACL-RSI compared with participants who scored above 56 points (table 2).

### Multivariable analyses

Due to few events (n=25), no predictive multivariate model could be created.

## Discussion

The main finding in this study was that lower levels of self-efficacy and psychological readiness to RTS 18 months after ACLR were associated with being PyI, 5–8 years after ACLR. Systematic reviews have repeatedly reported that psychological factors play an important role in RTS.^10 18 32^ For example, patients who RTS 12 months after ACLR report a higher level of knee-related self-efficacy, as well as psychological readiness to RTS, early in the rehabilitation compared with those that do not RTS.^8 18 30^ The associations between PI and knee-related self-efficacy and psychological readiness in the present study suggest that psychological factors could also help clinicians to identify individuals that run a higher risk of being PyI 5–8 years after ACLR. However, the AUC values of the models adjusted for patients’ sex ranged between 0.66 and 0.67, indicating that these predictions must be regarded with caution despite their potential influence on clinical practice.

Previous research has demonstrated that individuals who have undergone ACLR tend to be less PyA compared with healthy controls.^16 33^ In Sweden, a significant proportion (34%) of individuals between the ages of 16 and 64 fail to meet the WHO’s recommended guidelines of 150–300 min of physical activity a week.^7^ In the present study, only 14% were classified as PyI 5–8 years after ACLR. While this outcome is encouraging in terms of reducing non-communicable disease risk, it should be noted that study participants had a high level of preinjury physical activity, as evidenced by their Tegner scores, where about three in four were involved in knee-strenuous sport prior to their injury. For this reason, the proportion of PyI individuals in the present study group must still be considered too high. Furthermore, the proportion of PyI participants was almost two times larger in the group of participants who scored below 57.1 on the subscale of symptoms on the KOOS (table 2). Whether individuals who perceive more symptoms are less PyA or whether fewer PyA individuals develop more symptoms still remains to be determined. However, given the increased risk of developing symptomatic knee OA^34 35^ and overweight/obesity^12 15^ after ACL injury, it is essential for these patients to maintain physical activity throughout their lifetime.

### Limitations

This is one of the first studies evaluating rehabilitation-specific factors associated with PI in individuals after ACLR. In addition, few other studies have followed individuals after ACLR as long as 5–8 years with respect to PA. The strengths of this study include the prospectively collected data and the use of common and rigorously appraised questionnaires. However, the self-reported level of PA represents a limitation: it tends to be overestimated compared with objectively collected data.^36^ As a result, the prevalence of PI in the present study might be even higher. On the other hand, data were collected in February 2021, during the COVID-19 pandemic lockdown, which might have influenced the participants’ levels of PA. Additionally, only 25 participants were classified as PyI and, consequently, no multivariable adjusted model could be created. As a result, no conclusions about the associations between the dependent and the independent variables together were drawn. Correlations between the independent psychological variables used in this study have been reported previously^25 37 38^ and are expected, as the K-SES and the ACL-RSI were developed for similar patient groups and are designed to measure similar aspects.^37 38^ Moreover, the fact that the 41% of the eligible participants lost to follow-up were significantly younger and had a higher level of preinjury sport (Tegner>6) suggests a potential risk of bias. Additionally, as the frequency of exercise and sport is reported to decrease with age,^39^ the proportion of PyI may not be as high as 15% in the general ACL population. Further, as data regarding participants’ rehabilitation and PA between the follow-ups were not available, potential effects on the long-term outcomes cannot be ruled out. Additionally, there were no reliable data on concomitant injuries (eg, meniscus lesions) or postoperative knee complications. Therefore, we cannot rule out the possibility that these factors may have influenced psychological readiness to RTS or that PyI patients may have experienced more severe concomitant injuries or postoperative complications compared with PyA patients.

### Clinical implications and future research

The associations between PI and knee-related self-efficacy and psychological readiness to RTS suggest the importance of considering psychological factors in the long-term perspective of maintaining an active lifestyle. To facilitate the identification of individuals at risk of becoming PyI and hopefully prevent PI, healthcare professionals are recommended to evaluate and, if needed, help to enhance psychological factors throughout the entire rehabilitation. For instance, by implementing goal-setting,^40 41^ imagery,^41 42^ modelling^43^ and arousal control^41^ as a part of the rehabilitation.

The causal relationship between psychological outcomes and PI could be evaluated in future randomised controlled trials.

## Conclusion

Lower levels of knee-related self-efficacy and psychological readiness to RTS, 18 months after ACLR, were associated with PI 5–8 years after surgery.

## Data Availability

Data are available upon reasonable request. The dataset used and/or analysed is available from the corresponding author on request.
