# Supplementary material for: Physical inactivity 5–8 years after anterior cruciate ligament reconstruction is associated with knee-related self-efficacy and psychological readiness to return to sport
Source: BMJ Open Sport Exerc Med. 2023 Nov 14;9(4):e001687. doi: 10.1136/bmjsem-2023-001687 (PMC10649614; doi:10.1136/bmjsem-2023-001687)
Supplement: Supplementary data [file bmjsem-2023-001687supp001.pdf]

## The Swedish National Board of Health and Welfare physical activity questions

During a regular week, how much time do you spend exercising on a level that makes you short winded, for example running, fitness class or ball games?

- 0 minutes
- Less than 30 minutes
- 30 — 60 minutes (0.5 — 1 hour)
- 60 — 90 minutes (1— 1.5 hours)
- 90 — 150 minutes (1.5 — 2.5 hours)
- 150 — 300 minutes (2.5 — 5h)
- More than 300 minutes (>5 hours)

2. During a regular week, how much time are you physically active in ways that are not exercise, for example walks, bicycling or gardening? Add together all activities lasting at least 10 minutes.

- 0 minutes
- Less than 30 minutes
- 30 — 60 minutes (0.5—1 hour)
- 60 — 90 minutes (1—1.5 hours)
- 90 — 120 minutes (1.5 — 2 hours)
- More than 120 minutes (>2 hours)

Olsson SJ, Ekblom O, Andersson E, Borjesson M, Kallings LV. Categorical answer modes provide superior validity to open answers when asking for level of physical activity: A cross-sectional study. *Scand J Public Health*. Feb 2016;44(1):70-6. doi:10.1177/1403494815602830
